# Editorial: Emerging and re-emerging viral zoonoses

**DOI:** 10.3389/fmicb.2022.978259

**Published:** 2022-08-18

**Authors:** Naveen Kumar, Vladimir N. Uversky, Shailly Tomar, Kenneth S. M. Li, Keith Chappell, Susanna K. P. Lau

**Affiliations:** ^1^Zoonotic Diseases Group, Indian Council of Agricultural Research-National Institute of High Security Animal Diseases, Bhopal, India; ^2^Department of Molecular Medicine, Morsani College of Medicine, University of South Florida, Tampa, FL, United States; ^3^Institute for Biological Instrumentation of the Russian Academy of Sciences, Federal Research Center “Pushchino Scientific Center for Biological Research of the Russian Academy of Sciences, ” Pushchino, Russia; ^4^Department of Biosciences and Bioengineering, Indian Institute of Technology, Roorkee, India; ^5^Department of Microbiology, Li Ka Shing Faculty of Medicine, The University of Hong Kong, Pokfulam, Hong Kong SAR, China; ^6^School of Chemistry and Molecular Biosciences, The University of Queensland, Brisbane, QLD, Australia; ^7^Australian Institute of Bioengineering and Nanotechnology, The University of Queensland, Brisbane, QLD, Australia

**Keywords:** emerging zoonotic viral infections, cross-species transmission, evolutionary dynamics, host-pathogen interactions, molecular epidemiology, antivirals

Since the beginning of the twenty-first century, a number of novel emerging and re-emerging viruses, largely of zoonotic origin, have spread from wildlife reservoirs to human population, instigating deadly outbreaks of global concern (Karesh et al., 2012). A variety of factors can contribute to the emergence of a new zoonotic disease (Allen et al., 2017; Sikkema and Koopmans, 2021), including the following:

- Anthropogenic factors, such as illegal wildlife trade, intensive urbanization and expanding populations, and increasing global travel and trade;
- Environmental factors, including climatologic and ecologic changes;
- Emergence of viruses with altered genetic and biological characteristics;
- Use of laboratory animals in biomedical research.

This Research Topic, titled “*Emerging and Re-emerging Viral Zoonoses*,” included recent updates on emerging and re-emerging zoonotic viruses. It covers the newly discovered Orf virus transmission route, the evolution dynamics of the human Cowpox virus and Influenza A H5N8 virus, the antiviral activity of a food additive against Coxsackievirus group B, the role of migratory birds in *Deltacoronavirus* (δ-CoV) transmission, a rabies virus-based pseudovirus neutralization assay for the detection of neutralizing antibodies against Marburg virus, the epidemiology and pathogenicity assessment of ferret badger-associated rabies viruses, and the role of long non-coding RNA (lncRNA) nuclear-enriched abundant transcript 1 (NEAT1) in limiting Hantaan virus propagation.

For a successful perpetuation of zoonotic viruses, they must be efficient in crossing the species barrier in order to enter, replicate, and disseminate within a suitable host, and/or be transmitted to the same or a new host *via* different transmission routes. Therefore, understanding the transmission routes of viruses, whether direct or indirect, as well as their molecular evolutionary dynamics, forms an integral and primary component in formulating the guidelines for effective control and prevention of viral zoonoses, as well as assessing the public health risks. In contrast to the most frequently documented method of transmission, which involves direct contact with infected animals or fomites, Ma et al. identified the saliva and milk of asymptomatic goats as a potential novel source of Orf virus infection in humans. By using comprehensive genome and co-evolutionary approaches, Chu et al. highlighted the potential contribution of migratory birds to the transmission of δ-CoVs and forecasted frequent host-switching events among birds as well as interspecies transmission to a few mammalian species. Similarly, Ye et al. showed how wild birds contributed to the spread of H5N8 viruses among poultry, and how several reassortment events controlled the evolutionary dynamics of influenza viruses.

Furthermore, in order to overcome host selective pressures and adapt to a new host, viruses preferably undergo recombination, reassortment or acquire adaptive mutations. This allows for the generation of new genetic variants with altered transmissibility, virulence, and host switching capacity (Muñoz-Moreno et al., 2019; Kumar et al., 2021). In line with these considerations, Diaz-Cánova et al. demonstrated a Norwegian human Cowpox virus (CPXV) isolate carrying multiple genes from orthopoxviruses that originated from the Old World, including *Ectromelia virus* (ECTV) and *Vaccinia virus*, and North America, *Alaskapox virus* (AKPV), through multiple reassortment events. Four distinct lineages of ferret badger-associated rabies viruses with minimal pathogenicity in mice, beagles, and humans were found in China in a longitudinal analysis by Miao et al. These studies emphasize the importance of continuous monitoring of virus genetic variants and assessing the associated public health risks.

Significant progress has been made toward delivering potent vaccines and antiviral drugs (through drug repurposing) in short span of time. However, in order to effectively combat future pandemics, it is essential to initiate proactive preparedness programs at the global level. These programs should enhance the capacity for surveillance, rapid testing of available and new antiviral drugs, as well as generation of safe, efficacious and affordable vaccines (Belay et al., 2017; WHO, 2022). Contributing to these proactive efforts, Bi et al. reported a rabies virus-based pseudovirus neutralization assay for detecting the neutralizing activity of antibodies against Marburg virus and showed that this assay carried selective advantages in comparison to the traditional lentivirus-based pseudovirus neutralization assay. The proposed system, with the use of three pseudoviruses covering the two distinct Marburg virus lineages, offers a robust, safe and economical alternative for the quick assessment of vaccine efficacy and development of therapeutic antibodies/drugs. As a possible therapeutic target for the clinical management of severe hemorrhagic fever with renal syndrome, Yang et al. elucidated the function and molecular mechanism of lncRNA NEAT1 in controlling Hantaan virus propagation. Ethyl 3-hydroxyhexanoate (EHX), a volatile substance present in fruits and food additives, was shown by Olasunkanmi et al. to have antiviral activity against Coxsackievirus group B (CVB) both *in vitro* and *in vivo*. These studies offer the safe therapeutic avenues for the development of antiviral drugs for emerging viral infections.

Lessons learned from the emerging and re-emerging zoonotic viruses underscore the need for a more holistic and integrated strategy to averting future pandemics taking into the account of the health of humans and animals, and their shared environment; i.e., “one health approach.” The “one health approach” may be challenging to implement practically given the dramatic changes in the anthropogenic and environmental factors. However, if we implement large-scale intensive genomic surveillance programs along with the metagenomic surveillance at the animal-human interface, limit ecological space sharing with wildlife, and raise stakeholder awareness of “one health,” we can reduce the risk of future zoonotic spillover events. This Research Topic demonstrates the diverse spectrum of emerging and re-emerging viral zoonoses, and the need for understanding the evolutionary dynamics of viruses, complex host-pathogen interactions, and effective prophylactic measures. These aspects will ensure our preparedness for future pandemics.

## Author contributions

All of the listed authors contributed significantly to the work and gave their consent for publishing. All authors contributed to the article and approved the submitted version.

## Conflict of interest

The authors declare that the research was conducted in the absence of any commercial or financial relationships that could be construed as a potential conflict of interest.

## Publisher's note

All claims expressed in this article are solely those of the authors and do not necessarily represent those of their affiliated organizations, or those of the publisher, the editors and the reviewers. Any product that may be evaluated in this article, or claim that may be made by its manufacturer, is not guaranteed or endorsed by the publisher.
